# Hotspot analysis of COVID-19 infection using mobile-phone location data

**DOI:** 10.1007/s10015-022-00830-2

**Published:** 2022-11-27

**Authors:** Yu Kimura, Tatsunori Seki, Satoshi Miyata, Yusuke Arai, Toshiki Murata, Hiroyasu Inoue, Nobuyasu Ito

**Affiliations:** 1 IT-OT Innovation Division, SoftBank, 1-7-1 Kaigan, Minato-ku, Tokyo Japan 105-7529; 2 RIKEN Center for Computational Science, 7-1-26 Minatojima-minami-machi, Chuo-ku, Kobe, Hyogo Japan 650-0047; 3 Graduate School of Information Science, University of Hyogo, 7-1-28 Minatojima-minami-machi, Chuo-ku, Kobe, Hyogo Japan 650-0047

**Keywords:** COVID-19, Effective reproduction number, Hotspot analysis, Mobile phone data

## Abstract

Restrictions on outdoor activities are required to suppress the COVID-19 pandemic. To monitor social risks and control the pandemic through sustainable restrictions, we focus on the relationship between the number of people going out and the effective reproduction number. The novelty of this study is that we have considered influx population instead of staying-population, as the data represent congestion. This enables us to apply our analysis method to all meshes because the influx population may always represent the congestion of specific areas, which include the residential areas as well. In this study, we report the correlation between the influx population in downtown areas and business districts in Tokyo during the pandemic considering the effective reproduction number and associated time delay. Moreover, we validate our method and the influx population data by confirming the consistency of the results with those of the previous research and epidemiological studies. As a result, it is confirmed that the social risk with regard to the spread of COVID-19 infection when people travel to downtown areas and business districts is high, and the risk when people visit only residential areas is low.

## Introduction

Over the past 2 years, it has been globally accepted that restrictions on outdoor activities are required to reduce the number of COVID-19-positive cases [1]. Currently, studies on distinguishing “high-risk” activities have been conducted. For instance, Bale et al. [2] simulated the dispersion mechanism between droplets an aerosol using a supercomputer. These studies provide important insights into personal risks; however, studies that provide macro insights on social risks are required to monitor and control the pandemic.

Although several studies on personal risks and behaviors have already been reported, analytical methods to study the relationship between social activity and infection risk levels have not been established. Ito [3] developed a disease spread simulation model and GPS data miner, which operates with a supercomputer and thus enables the simulation of various scenarios in a short period time. Despite the potential of these systems, as GPS location data tend to contain relatively large statistical errors, owing to the small population size, this analytical method may only be applicable to urban areas. Nakanishi et al. [4] observed night-time population data derived from the mobile phone base station logs of the Tokyo metropolitan areas and reported an increase in the effective reproduction number 3 weeks after the night-time population increased. Ishida et al. [5] suggested a mathematical method to extract areas where the night-time population was correlated with the number of new positive cases in that city 11 days later based on the night-time population count obtained from mobile-phone base station logs. As the population size of the location data of mobile phones is larger than that of GPS-derived data, the statistical error is smaller. However, for the night-time population in residential areas is considered as the sum of the number of people staying at home and those going out within the residential areas, it may be difficult to discuss the relationship between the infection risk and social activity levels in residential areas in the same way as in downtown areas. Ishida et al. [5] considered areas only where the peak population was more than 2000 in a 250-m^2^ area, whereas Nakanishi et al. [4] focused on metropolitan areas. Using the influx population data, the effects of people travelling in downtown areas, business districts, and residential areas on the spread of the COVID-19 are comparable, and thus, we can discuss the risk factor of people going out. As people tended to avoid going to downtown areas, to try not to go to business districts, and to stay home during the periods of the state of emergency, and trend defining the number of new COVID-19 cases declined in those periods, we set the following hypothesis: influx population in downtown areas is strongly related to the spread of COVID-19 infection whereas in business districts, the relation is moderate, and in residential areas the influx population is least related. This hypothesis is to be proved in Sect. 3.

We have extracted influx population data (mentioned in Sect. 2) from the system logs of mobile phone base stations of SoftBank. Using these data, the relationship between the infection risk and social activity levels in downtown areas, business districts, and residential areas can be investigated with small statistical errors.

## Data and methods

### Influx population data

Location information data used in this study to estimate the influx population of 500-m^2^ meshes (of which the side length is half of that of the standard mesh) were extracted from the system logs of the mobile phone base stations of SoftBank, which is one of the lines of Zenkoku-Ugoki-Tokei [6]. A “mesh” is a grid square; more information is available on the Research Institute for World Grid Squares website [7].

**Fig. 1:**
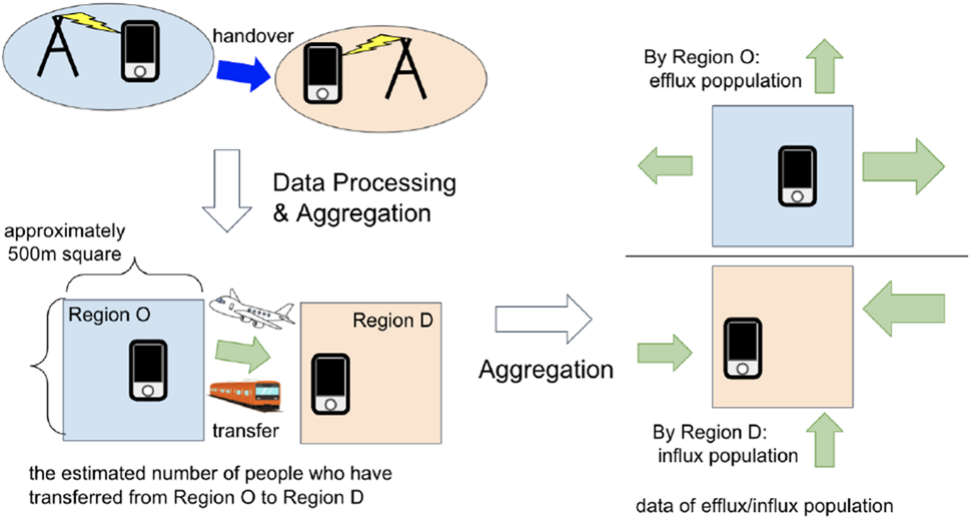
Outline of the data processing to obtain the influx population data

Each mobile phone in service registers its current location (Fig. 1, upper left) regardless of whether the user communicates (e.g., makes or receives phone calls) or not. As it is impossible to predict whether (or what) the user has communicated from this location information, the data processed through this system log do not violate the privacy of communications. Please visit our website [8] to obtain more information about our policies with regard to the use and application of customer data.

As we can use the system logs of the users of SoftBank whose consent is acquired through statistical processes and aggregations, we obtain “Origin to Destination data” (later, we refer to as “OD data”), the data of the estimated number of people who have transferred from a specific region (O) to another specific region (D) (Fig. 1, bottom left). Passers are removed from the count in this process. The numbers in the OD data are the expected numbers of people including non-SoftBank users. By simply calculating the sum of the OD data table without grouping based on the O region column, we obtained the influx population data (Fig. 1, right). As these are well-anonymized statistical data, individual customers are hardly identified.

The D regions and the period of the influx population data used in this study were 500 m-m^2^ meshes in specific areas of Tokyo (Table 2 in the appendix section); the duration considered in this study is from November 1, 2020, to February 13, 2021. Although downtown areas and business districts tend to exist in the center area of Tokyo, whereas residential areas tend to exist in the surrounding areas, we have selected D areas from multiple cities so that geographical bias does not significantly affect the analysis. For example, Toyosu is located in the southeast part of Tokyo and is closer to downtown areas and business districts rather than the other residential areas (Fig. 9 in the appendix section). In the later chapter, it has been confirmed that the influx population data have exhibited a similar time series change among the residential areas.

As there are a large number of users, the statistical error is expected to be smaller than that of GPS-derived data. In addition, the influx population data includes the resident city column, and we can compare new COVID-19 cases reported in 23 regions of Tokyo and the influx population data of the residents for the same regions.

### Data of new COVID-19 cases

The data of the number of new COVID-19 cases were obtained from the portal site of the Bureau of Social Welfare and Public Health [9]. In this study, we used the number of new COVID-19 cases identified in the 23 regions of Tokyo.

### Analysis method

We converted the data of the new COVID-19 cases into daily series data of effective reproduction numbers using a simplified formula suggested by Nishiura et al. [10].

1 $$s\hat{R}\left[ d \right] = \left( {\frac{{\mathop \sum \nolimits_{j = 1}^{T} C\left[ {d - T + j} \right]}}{{\mathop \sum \nolimits_{j = 1}^{T} C\left[ {d - 2T + j} \right]}}} \right)^{{\left( {g/T} \right)}} ,$$

where $\hat{R}$[*d*] and *C*[*d*] are the effective reproduction number and the number of cases reported on day *d*, respectively. The parameters *g* and *T* denote the mean generation time and length of the reporting interval, respectively. The mean generation time is almost equal to the serial interval time, which Nishiura et al. [11] estimated as 4.7 ± 2.9 days. The reporting time *T* has been set to ~ 7 days, as Pavlicek et al. [12] reported that the number of new cases in Japan oscillates with a cycle of 7 days. In this study, we set *g* = 5 and *T* = 7 as the significant figures as one digit. The benefit of adopting this formula is that as the formula includes the number of cases reported on day *d,* we do not need the number of cases infected on day *d*, which Ishida et al. [5] manually estimated in their study. As the influx population data oscillates with a 7-day cycle (from Monday to Sunday), in the analysis, the effective reproduction number and influx population were averaged based on weeks.

The data of new COVID-19 cases and influx population are used only for people living in Tokyo. The data of November 1, 2020, to February 13, 2021, were collected, and the effects of vaccination were not deemed necessary to be considered. From January 7, 2021, to March 21**,** 2021, a state of emergency was announced.

## Results and discussion

### Correlations between the influx population and effective reproduction number considering time delay

**Fig. 2:**
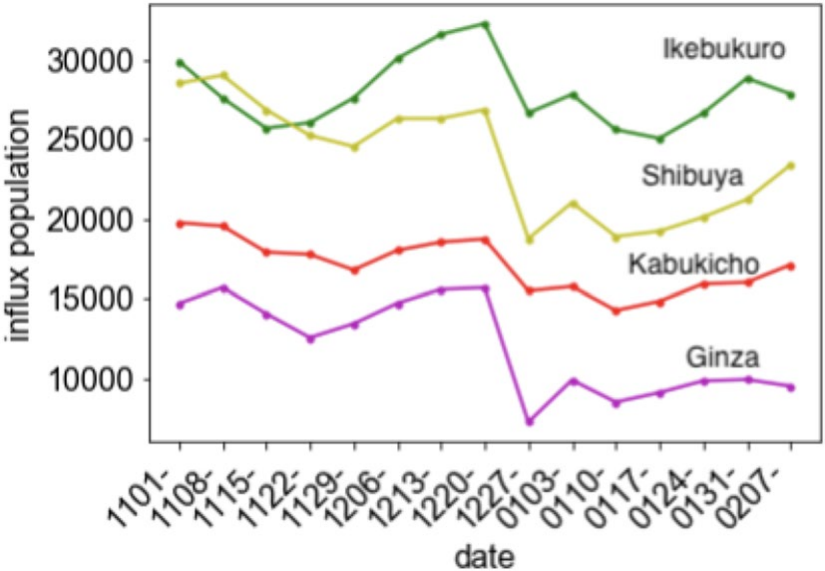
Time series variation of the weekly averages of the influx population in downtown areas from November. 1, 2020, to February. 13, 2021

**Fig. 3:**
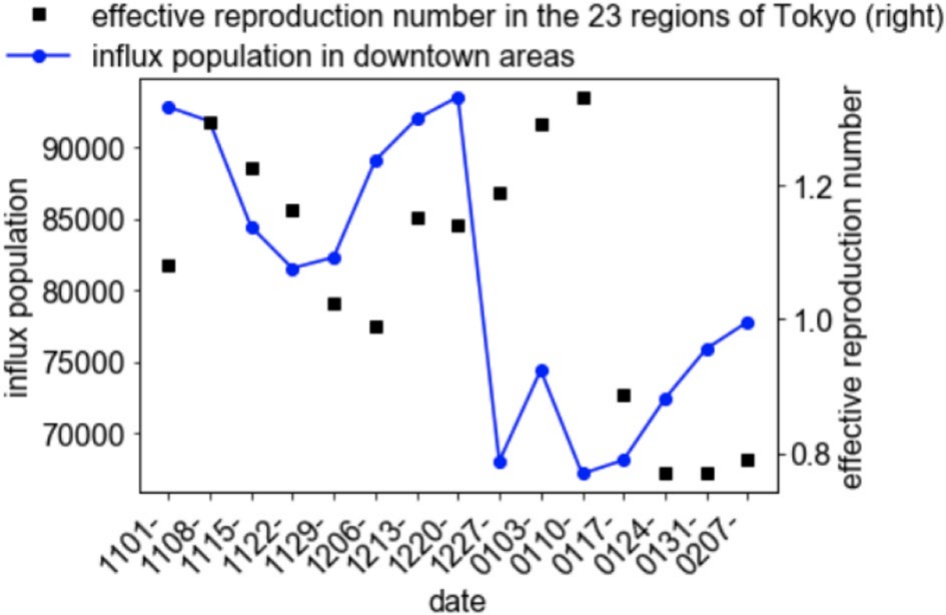
Time series variation of the sum of the averages of the influx population in downtown areas and the effective reproduction number

The trends of the influx population in the downtown areas are similar to each other, although the amount of the diminution based on the announcement of a state of emergency from the week of “0103” differs slightly (Fig. 2). The time series variation of the sum of these influx populations is quite similar to that of the effective reproduction number (Fig. 3) assuming that the effective reproduction number is delayed for ~ 3 weeks. Considering this assumption, the correlations were calculated with a time delay of 1, 2, 3 and 4 weeks (Table 1).

**Table 1:** Correlation coefficients with a varying delay period

Type	No delay	1 week delay	2 weeks delay	3 weeks delay	4 weeks delay
Downtown area	0.19	0.51	0.64	0.86	0.61
Business districts	0.20	0.42	0.48	0.75	0.56
Residential area	− 0.37	0.14	0.21	0.28	− 0.10

**Fig. 4:**
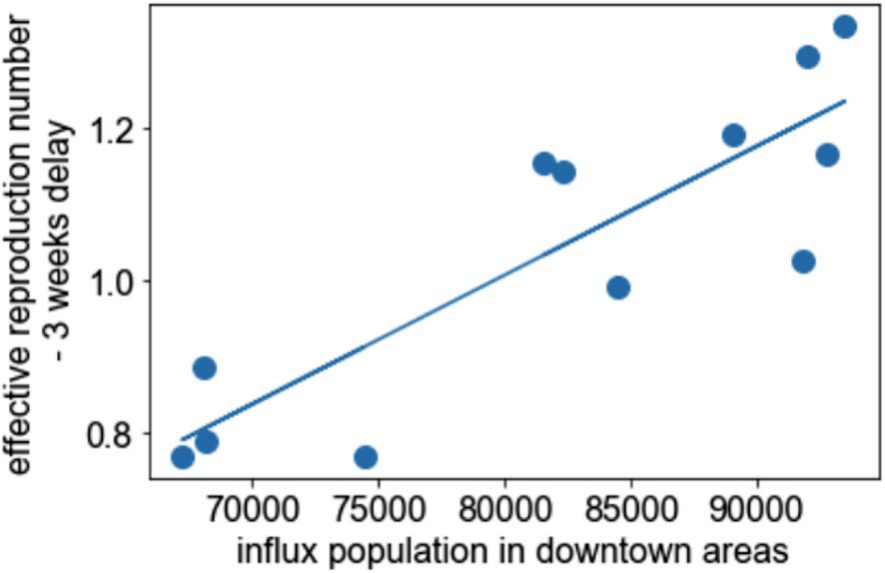
Scatter plot of the average of the influx population in downtown areas and the effective reproduction number with a time delay of 3 weeks

If we assume that there is a time delay of 3 weeks, the effective correlation number and the influx population in downtown areas are strongly correlated (= 0.86), as shown in the scatter plot (Fig. 4). The linear regression coefficient was 1.7 × 10^(−5)^.

**Fig. 5:**
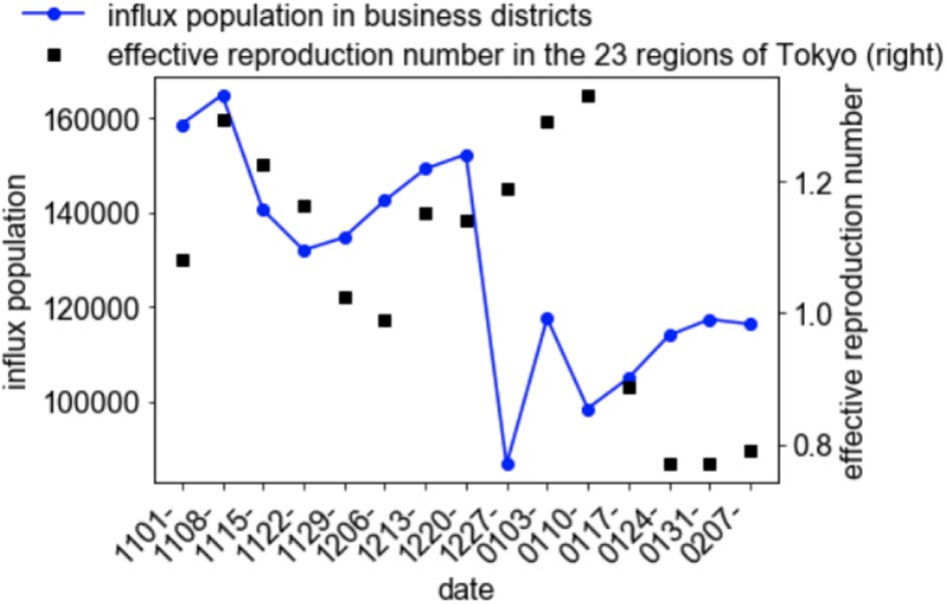
Time series variation of the sum of the averages of the influx population in the downtown areas and effective reproduction number

**Fig. 6:**
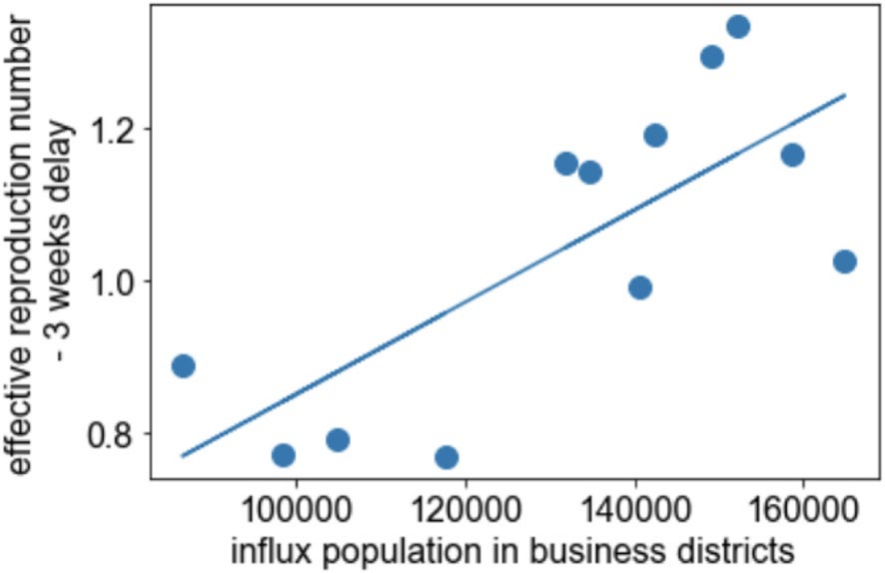
Scatter plot of the average of the influx population in business districts and effective reproduction number with a time delay of 3 weeks

**Fig. 7:**
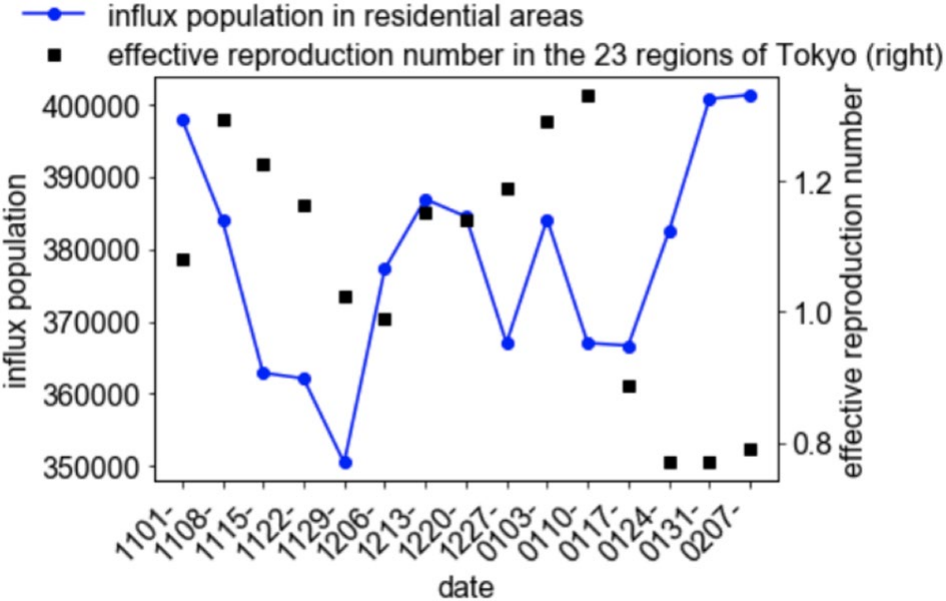
Time series variation of the sum of the averages of the influx population in residential areas and effective reproduction number

**Fig. 8:**
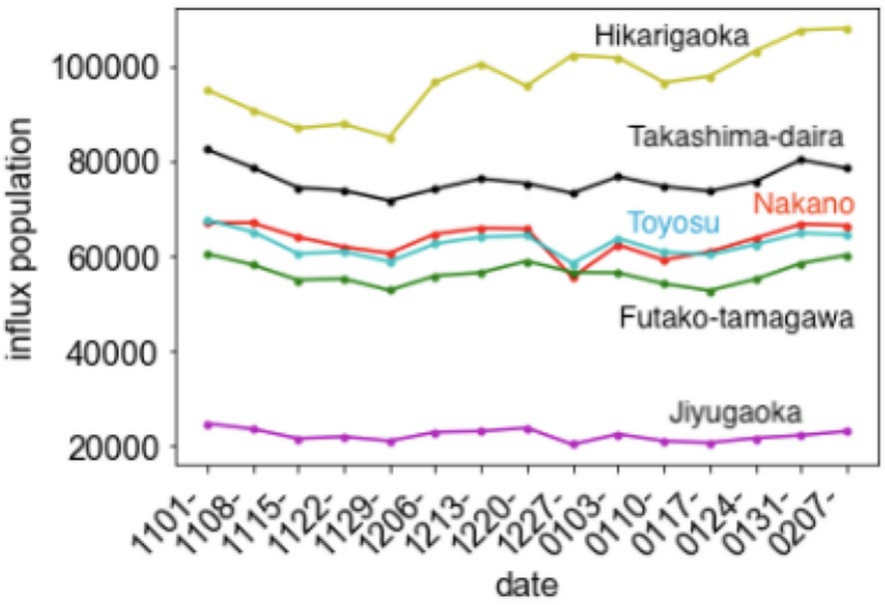
Time series variation of the weekly averages of the influx population in the residential areas

**Fig. 9:**
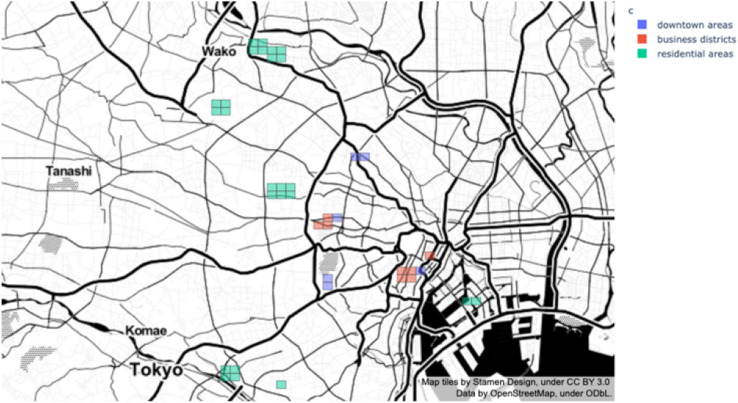
Meshes of the areas plotted on a map

The time series variation of the sum of the influx population in business districts exhibited a similar trend (Fig. 5) with a mild correlation (= 0.75, if time delay for 3 weeks is assumed), and the linear regression coefficient under the assumption of time delay for 3 weeks was 6.0 × 10^(−6)^ (the scatter plot is Fig. 6). Meanwhile, the residential areas did not exhibit any correlation (= 0.29 or smaller, Table 1) (Fig. 7). This is not caused by the feature cancellation of the summation, because the trends of the influx population in residential areas are quite similar (Fig. 8).

### Discussion

The influx population of downtown areas and business districts correlated with the effective reproduction number assuming that there is a delay of 3 weeks (Table 1). This result is consistent with the report of Nakanishi et al. [4] on the night-time population and is almost twice as long as the period that Ishida et al. [5] assumed from the incubation time and days taken to report a case. The delay of 3 weeks is as a few times as long as the generation time of COVID-19 reported by Nishiura et al. [11]; therefore, this cannot be defined as a single incubation time. It may take a few steps of infections until the effective reproduction number, which is a macro indicator rise. According to [13], this may be because it takes some time to spread and to raise the effective reproduction number from when a few people are infected at hotspots, and the night-time population may be related to the infections at those hotspots. This hypothesis is still unclear; therefore, the delay of 3 weeks should be explored considering the epidemiological side to discuss whether there are any steps of COVID-19 distribution, or if it is caused by an averaging effect. The duration of 3 weeks may represent the expected number of generations until a super-spreader appears in the infection chain. Based on the data analysis of people going out, we confirmed a relation between the effective reproduction number and influx population data similar to that of night-time population data in downtown areas.

The result of this study shows that the influx population in downtown areas strongly correlates (> 0.8) with the effective reproduction number, which is similar to the results of other studies [3–5]. In this study, we have used “influx” population data, so that the result implies that the number of people going out within the residential area does not considerably correlate with the effective reproduction number so much, although the period we focused on was before the vaccinations.

The regression coefficient and correlation were larger in downtown areas than in business districts. This result indicates that the influx population in downtown areas may have a more significant effect than that in business districts. The correlation between the effective reproduction number and influx population in business districts may need further investigations to understand whether this is caused by the correlation with the influx population of downtown areas, or whether social activity in business districts features different risks to social activities in downtown areas. This study does not mention the personal risks of people travelling within the residential areas; however, it mentions social risks when people travel to downtown areas or business districts.

### Main results

As we are using different types of data of people flow, we confirmed that our results are consistent with those of the previous studies [3–5]: an increase in the night-time population in the downtown areas was observed followed by the spread of the COVID-19 infection after a few weeks.

Then, we explored to our main hypothesis: the influx population in the downtown areas exhibits the highest correlation with the spread of COVID-19 infection, whereas the influx population in the business districts exhibits moderate correlation, and the influx population in the residential areas exhibit the least correlation. This is the novel point of this study, because the hypothesis becomes difficult to prove by night-time population data.

## Summary and perspectives

In this study, we reported the correlations between the effective reproduction number and influx population in 500-m^2^ meshes in specific areas of Tokyo. In future studies, we plan to group meshes based on the features of the time series variation of the influx population data instead of manual labeling, as implemented in this study. We expect these studies to enable us to monitor the social risk of pandemics based on changes in the influx population in downtown areas and business districts. Then, we will explore whether and how COVID-19 infection spreads from hotspots.

## Data Availability

The data that support the findings of this study are licensed from SoftBank where the authors conduct this study and are available from Zenkoku-Ugoki-Tokei [6] for a fee. Because of the restriction in the licensing agreement with SoftBank, the authors have no right to disclose the data publicity.

## Appendix

See Table 2 and Fig. 9

**Table 2:** Mesh codes of areas considered in this study

Type	City	Mesh code
Downtown area	Shibuya	533935952, 533935954
Downtown area	Kabukicho	533945363
Downtown area	Ikebukuro	533945773 533945774
Downtown area	Ginza	533946002
Business district	Kamiyacho	533935994
Business district	Nishi-shimbashi	533936903, 533946001
Business district	Toranomon	533945092
Business district	Shinjuku-nishi	533945351, 533945352, 533945354
Business district	Yaesu	533946111
Residential area	Jiyugaoka	533935233
Residential area	Futako-tamagawa	533935301, 533935302, 533935303, 533935304
Residential area	Toyosu	533936831, 533936832
Residential area	Nakano	533945522, 533945524, 533945531, 533945532, 533945433, 533945434
Residential area	Hikarigaoka	533954094, 533954192, 533955003, 533955101
Residential area	Takashima-daira	533955414, 533955422, 533955423, 533955424, 533955431, 533955433, 533955512, 533955521
